# Recombinant protein production associated growth inhibition results mainly from transcription and not from translation

**DOI:** 10.1186/s12934-020-01343-y

**Published:** 2020-04-06

**Authors:** Zhaopeng Li, Ursula Rinas

**Affiliations:** 1 Leibniz University of Hannover, Technical Chemistry-Life Science, Callinstr. 5, 30167 Hannover, Germany; 2 Helmholtz Centre for Infection Research, Inhoffenstraße 7, 38124 Brunswick, Germany

**Keywords:** *Escherichia coli*, Recombinant protein production, Metabolic burden, Transcriptional burden

## Abstract

**Background:**

Recombinant protein production can be stressful to the host organism. The extent of stress is determined by the specific properties of the recombinant transcript and protein, by the rates of transcription and translation, and by the environmental conditions encountered during the production process.

**Results:**

The impact of the transcription of the T7-promoter controlled genes encoding human basic fibroblast growth factor (hFGF-2) and green fluorescent protein (GFP) as well as the translation into the recombinant protein on the growth properties of the production host *E. coli* BL21(DE3) were investigated. This was done by using expression vectors where the promoter region or the ribosome binding site(s) or both were removed. It is shown that already transcription without protein translation imposes a metabolic burden on the host cell. Translation of the transcript into large amounts of a properly folded protein does not show any effect on cell growth in the best case, e.g. high-level production of GFP in Luria–Bertani medium. However, translation appears to contribute to the metabolic burden if it is connected to protein folding associated problems, e.g. inclusion body formation.

**Conclusion:**

The so-called metabolic burden of recombinant protein production is mainly attributed to transcription but can be enhanced through translation and those processes following translation (e.g. protein folding and degradation, heat-shock responses).

## Background

The most common *E. coli*-based expression system is the bacteriophage T7 RNA polymerase and T7 promoter controlled gene expression combination. IPTG-induction of the chromosomally encoded bacteriophage T7 RNA polymerase leads to high level expression of T7 promoter-controlled genes and correspondingly high recombinant protein titers. High level recombinant protein production in *Escherichia coli* frequently leads to an impact on host cell metabolism detectable through growth retardation and generally known as “metabolic burden” [1, 2]. The factors contributing to the metabolic burden are multifaceted depending on the host/vector combination and on the properties of the encoded gene with its transcription and translation products as well as on the environmental conditions encountered during the production process [3–6]. The prime reasons for the protein production related metabolic burden have been attributed to protein translation and protein folding [1, 6] as well as to transcription and plasmid amplification related processes [7–9]. However, production of the same protein under identical conditions in the same host/vector combination but with single codon exchanges can change the metabolic perturbations considerably [10–13] suggesting that the phenomenon is even more complex than anticipated.

In this contribution we analyze the impact of T7-promoter-controlled recombinant gene expression on the host cell metabolism using two different model proteins, the difficult-to-fold human basic fibroblast growth factor (hFGF-2) with its high propensity to form inclusion bodies in *E. coli* and the easy-to-fold green fluorescent protein (GFP) mainly produced as soluble protein. For both examples we generated a set of expression vectors in which either the ribosome binding site (RBS) or the promoter region or both were removed in order to analyze if the plasmid itself, the transcription of the recombinant gene, the translation into the protein product and/or protein folding related processes have an impact on host cell metabolism.

## Results

### Different proteins induce different growth inhibitions

**Fig. 1:**
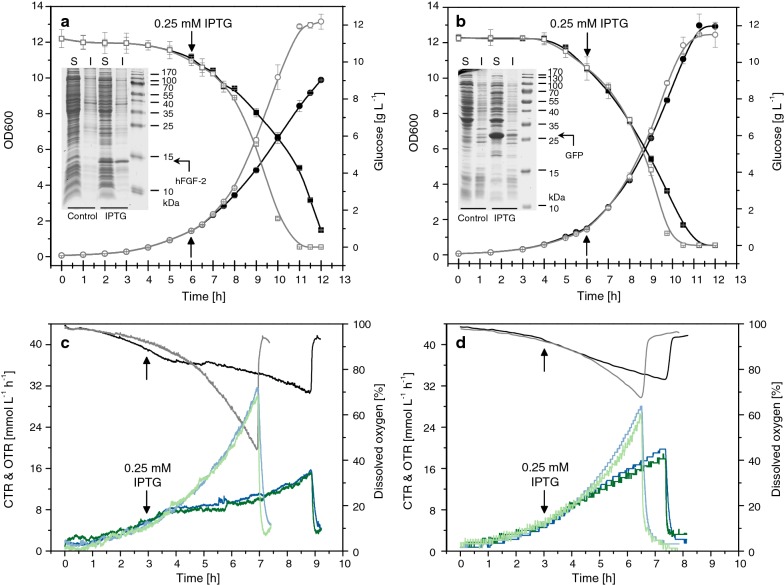
Growth inhibition resulting from the production of different recombinant proteins. Production of **a**, **c** hFGF-2 and **b**, **d** GFP using *E. coli* BL21 (DE3) as expression host was carried out in **a**, **b** Fernbach flasks with baffles and in **c**, **d** bioreactor batch cultures employing a defined mineral salt medium with glucose as carbon substrate. **a**, **b** OD600 (circles), glucose (squares) and SDS-PAGE analysis of target protein production (S: soluble, and I: insoluble cell protein of control and producing cells, the positions of target proteins are indicated) are shown. **c**, **d** Carbon dioxide (CTR, green lines) and oxygen transfer rates (OTR, blue lines) as well as dissolved oxygen concentrations (black and gray lines) are given. The arrows indicate the time point of IPTG addition to a final concentration of 0.25 mM in production experiments. Dark colored symbols and lines correspond to production experiments. Light colored lines and open gray symbols correspond to control experiments where IPTG addition was omitted. Data are presented as mean ± SD, n = 2

The production of recombinant proteins can impair cellular growth detectable through a slower increase in cell mass or even complete growth inhibition, slower carbon substrate uptake, and reduced respiratory activity. The extent of this inhibition is strongly influenced by the properties of the recombinant protein, a phenomenon noticeable when different proteins are being produced in the same host strain/expression system combination under identical production conditions (Fig. 1). For example, the production of hFGF-2, a protein partly produced as soluble protein and partly in form of inclusion bodies, leads to a strong impact on bacterial growth ([14–16], Fig. 1a). On the other hand, production of the green fluorescent protein (GFP), mainly produced as soluble protein in *E. coli*, impairs bacterial growth only marginally (Fig. 1b). Although this protein is produced in larger amounts at identical conditions compared to hFGF-2, production of GFP leads to a less severe decline of growth and metabolic activities (Fig. 1).

To distinguish if recombinant protein synthesis and/or transcription of the recombinant gene are responsible for the growth inhibitory effect a set of modified expression vectors was constructed. All expression vectors carried a gene encoding a recombinant protein (hFGF-2 or GFP) with the original T7-based production vectors containing in front of the target gene the T7 promoter and a ribosome binding site (RBS). One set of expression vectors was modified in such a way that transcription of the recombinant gene was allowed by IPTG addition but translation into the protein product omitted by removal of the RBS. In a second set of expression vectors the promoter region was removed to prevent transcription of the recombinant gene. And in a third set of expression vectors the promoter region as well as the RBS were removed.

### Already transcription of the recombinant gene causes growth inhibition

**Fig. 2:**
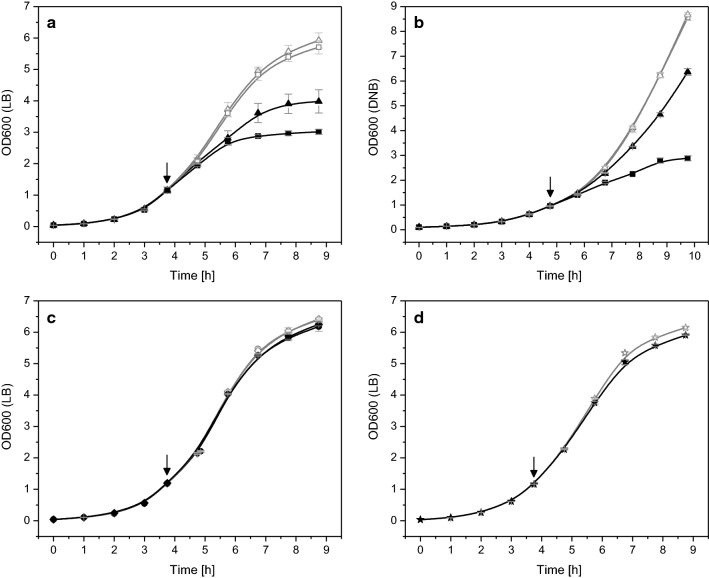
Already transcription of the recombinant gene causes growth inhibition. Growth properties of *E. coli* BL21 (DE3) with expression vectors carrying the gene encoding hFGF-2 in shake-flask cultures with **a** Luria–Bertani (LB) or **b** defined glucose-supplemented mineral salt medium. Growth of cells carrying the original expression vector with promoter region and with RBS (squares) and the expression vector with promoter region but without RBS in front of the hFGF-2 gene (triangles). **c** Growth of cells on LB medium carrying the expression vector without promoter region but with RBS (circles) and without promoter region and without RBS (diamonds). **d** Control cultures of cells without any expression vector growing on LB medium (stars). Full black symbols always correspond to IPTG induced cultures and open gray symbols to cultures where IPTG addition was omitted. The time-point of IPTG addition to a final concentration of 0.5 mM is indicated by an arrow. Data are presented as mean ± SD, n = 4

**Fig. 3:**
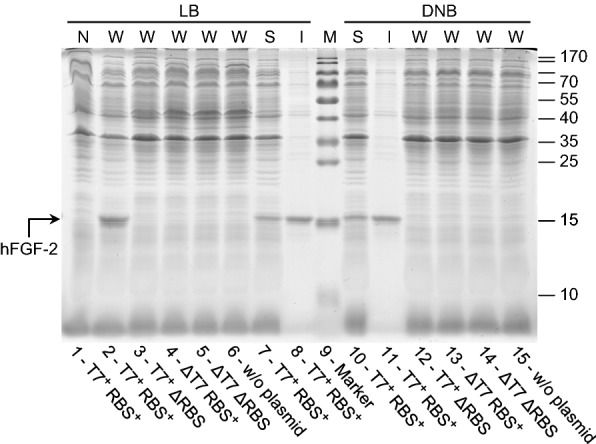
hFGF-2 production only detectable when promoter region and RBS are present. SDS-PAGE analysis of IPTG induced cells carrying the gene encoding hFGF-2 growing on LB medium (lanes 1–8) and glucose supplemented mineral salt medium DNB (lanes 10–15) collected 4 h after induction. Lane 1: N, T7^+^ RBS^+^ (non-induced control, total cell extract of cells containing the expression vector with promoter and with RBS in front of the hFGF-2 gene), lane 2: W, T7^+^ RBS^+^ (induced cells with promoter and with RBS), lanes 3, 12: W, T7^+^ ΔRBS (with promoter but without RBS), lanes 4, 13: W, ΔT7 RBS^+^ (without promoter but with RBS), lanes 5, 14: W, ΔT7 ΔRBS (without promoter and without RBS), lanes 6, 15: W, w/o plasmid (induced *E. coli* BL21(DE3) without expression vector), lanes 7, 10: S, T7^+^ RBS^+^ (soluble) and lanes 8, 11: I, T7^+^ RBS^+^ (insoluble cell fraction of induced cells containing the expression vector with promoter and with RBS). Lane 9: molecular mass marker, the position of hFGF-2 is indicated by an arrow. N: non-induced whole cell protein W: induced whole cell protein, S: induced soluble part, and I: induced insoluble part of whole cell protein

**Fig. 4:**
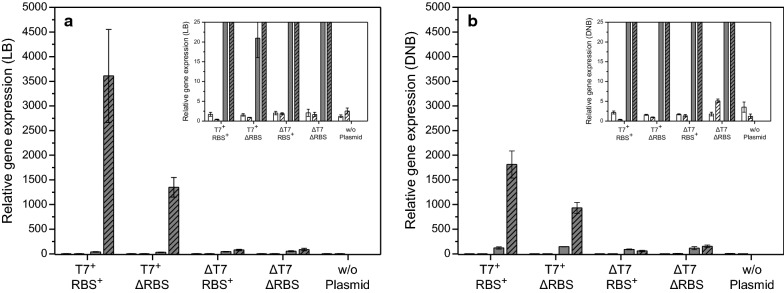
hFGF-2 transcript levels under inducing and non-inducing conditions. **a** Transcript levels in *E. coli* BL21(DE3) transformed with expression vectors carrying the gene encoding hFGF-2 in shake-flask cultures with **a** Luria–Bertani (LB) medium or **b** defined glucose-supplemented mineral salt medium (DNB). Trancripts of genes encoding the major elongation factor EF-Tu (white bars) and hFGF-2 (gray bars) are shown in non-induced cells (open bars) and 4 h after IPTG induction (hatched bars). T7^+^ RBS^+^: cells containing the expression vector with promoter and with RBS in front of the hFGF-2 gene; T7^+^ ΔRBS: with promoter but without RBS; ΔT7 RBS^+^: without promoter but with RBS; ΔT7 ΔRBS: without promoter and without RBS; w/o plasmid: *E. coli* BL21(DE3) without expression vector. The inserts depict the same data as the main figures only at different scale. Transcripts of *rrsH*, were used as internal standard and artificially defined as 10,000. Data are presented as mean ± SD, n = 4

Cells of *E. coli* BL21(DE3) transformed with these different types of plasmids carrying the gene encoding hFGF-2 were grown in shake flask cultures in complex Luria–Bertani medium as well as in defined glucose-supplemented mineral salt medium and cell growth was followed after IPTG addition (Fig. 2). Cells carrying the production vectors with intact promoter region and RBS revealed the expected growth inhibition in both types of media (Fig. 2a, b). Cells carrying the plasmids without RBS but intact promoter region also revealed growth inhibition upon IPTG addition showing that already enhanced recombinant gene transcription without recombinant protein synthesis negatively impacts cell growth (Fig. 2a, b). However, in both types of media the growth inhibition was intensified when hFGF-2 was produced. SDS-PAGE analyses verified the presence of hFGF-2 in induced cells carrying the production vector with intact promoter region and RBS and absence in those induced cells transformed with the expression vector where the RBS in front of the hFGF-2 gene was removed (Fig. 3). Compared to transcript levels of the *tufA* gene, encoding the major housekeeping protein and elongation factor EF-Tu, transcript levels of hFGF-2 were largely enhanced in both types of transformed cells, carrying the plasmid with intact promoter region with and without RBS, after induction in LB (Fig. 4a) as well as in defined medium (Fig. 4b). For example, in cells carrying the original expression vector and induced in LB medium hFGF-2 transcripts reached more than 2000 times higher levels than those from the *tufA* gene. Non-induced control cells and induced control cells transformed with the vectors where the promoter region in front of the hFGF-2 gene was removed did not produce hFGF-2 (Fig. 3) and only revealed basal transcripts levels of the plasmid-encoded hFGF-2 gene (Fig. 4). Induced control cells carrying expression vectors without intact promoter region and the induced host *E. coli* BL21(DE3) not carrying any expression vector revealed the same marginal almost undetectable growth inhibition compared to the non-induced cells (Fig. 2c, d) demonstrating that plasmid maintenance and constitutive expression of plasmid encoded (antibiotic resistance) genes do not lead to any growth inhibition. A marginal growth retardation can be attributed to induced expression and synthesis of the chromosomally encoded T7 RNA polymerase (Fig. 2d).

### Recombinant protein translation itself does not cause growth inhibition

**Fig. 5:**
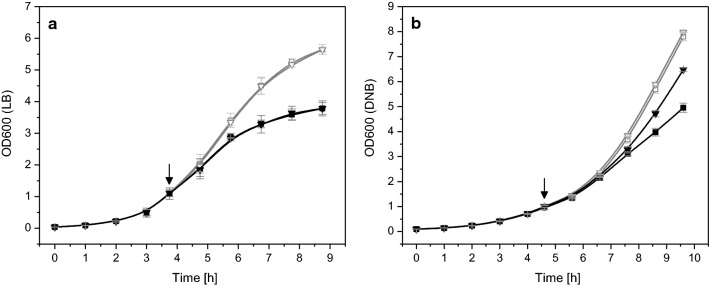
Recombinant protein translation itself does not impair cell growth but folding associated problems contribute to growth inhibition. **a** Growth properties of *E. coli* BL21(DE3) with expression vectors carrying the gene encoding GFP in shake-flask cultures with **a** Luria–Bertani medium or **b** defined glucose-supplemented mineral salt medium (DNB). Growth of cells carrying the original expression vector with promoter region and with RBS (squares) and the expression vector with promoter region but without RBS in front of the hFGF-2 gene (triangles). Full black symbols always correspond to IPTG induced cultures and open gray symbols to cultures where IPTG addition was omitted. The time-point of IPTG addition to a final concentration of 0.5 mM is indicated by an arrow. Data are presented as mean ± SD, n = 4

In another set of experiments cells carrying the GFP encoding production vectors with intact promoter region (with and without RBS) were grown in shake flask cultures in complex Luria–Bertani medium as well as in defined glucose-supplemented mineral salt medium and cell growth was followed after IPTG addition (Fig. 5). These experiments revealed for cells carrying the production vector with intact promoter region and RBS the expected growth inhibition in both types of media, but less pronounced compared to hFGF-2 producing cells (Fig. 5a, b versus Fig. 2a, b).

**Fig. 6:**
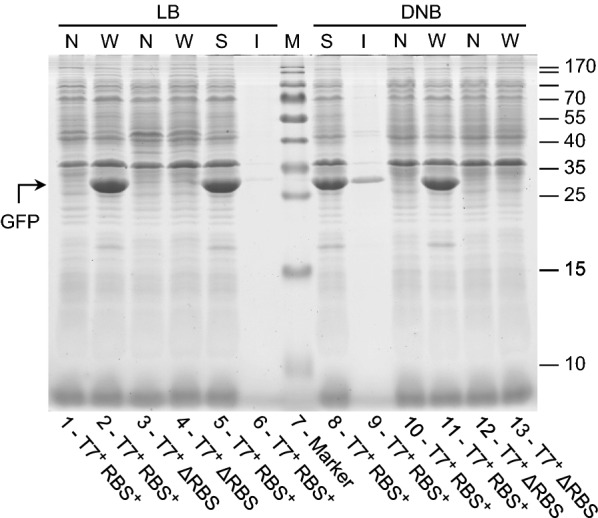
GFP only forms inclusion bodies during production on defined medium. SDS-PAGE analysis of IPTG induced cells carrying the gene encoding GFP growing on LB medium (lanes 1–6) and glucose supplemented mineral salt medium (lanes 8–13) collected 4 h after induction. Lanes 1, 10: N, T7^+^ RBS^+^ (non-induced controls, total cell extract of cells containing the expression vector with promoter and with RBS in front of the GFP gene), lanes 2, 11: W, T7^+^ RBS^+^ (induced cells with promoter and with RBS), lanes 3, 12: N, T7^+^ ΔRBS (non induced controls with promoter but without RBS), lanes 4, 13: W, T7^+^ ΔRBS (induced cells with promoter but without RBS), lanes 5, 8: S, T7^+^ RBS^+^ (soluble) and lanes 6, 9: I, T7^+^ RBS^+^ (insoluble cell fraction of induced cells containing the expression vector with promoter and with RBS). Lane 7: molecular mass marker, the position of GFP is indicated by an arrow. N: non-induced whole cell protein W: induced whole cell protein, S: induced soluble part, and I: induced insoluble part of whole cell protein

**Fig. 7:**
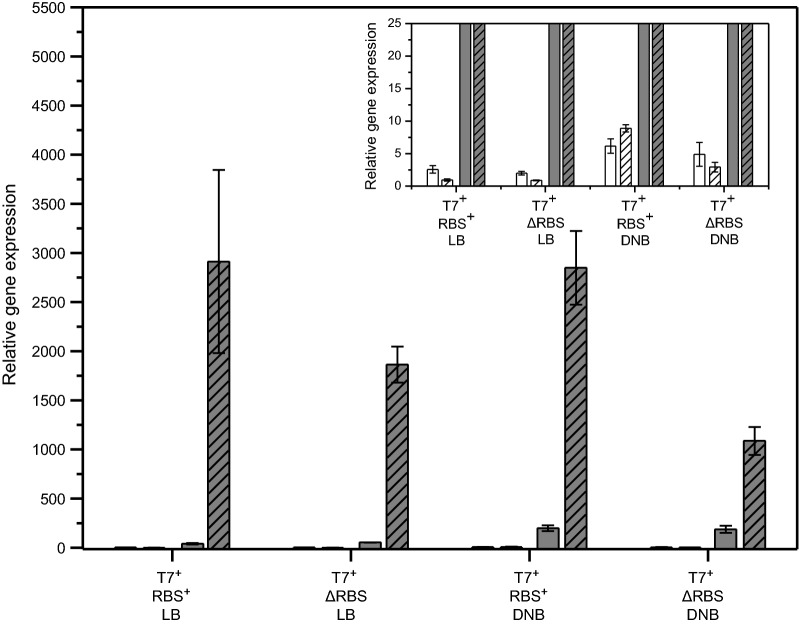
GFP transcript levels under inducing and non-inducing conditions. Transcript levels in *E. coli* BL21(DE3) transformed with expression vectors carrying the gene encoding GFP in shake-flask cultures with Luria–Bertani (LB) medium or defined glucose-supplemented mineral salt medium (DNB). Trancripts of genes encoding the major elongation factor EF-Tu (white bars) and GFP (gray bars) are shown in non-induced cells (open bars) and 4 h after IPTG induction (hatched bars). T7^+^ RBS^+^: cells containing the expression vector with promoter and with RBS in front of the GFP gene; T7^+^ ΔRBS: with promoter but without RBS. The insert depicts the same data set as the main figure only at different scale. Transcripts of *rrsH* were used as internal standard and artificially defined as 10,000. Data are presented as mean ± SD, n = 4

Interestingly, cells carrying the plasmid without RBS but intact promoter region in front of the GFP gene revealed the same growth inhibition in LB medium upon IPTG addition as those cells transformed with the plasmid having the intact promoter region as well as the RBS (Fig. 5a) suggesting that only recombinant gene transcription but not protein translation itself contributes to growth inhibition. The presence and absence of the recombinant GFP in induced cells carrying the expression vector with and without RBS was proven by SDS-PAGE analysis, respectively (Fig. 6) as well as the presence of elevated GFP transcript levels in both types of transformed cells upon induction in LB medium (Fig. 7). Thus, the data prove that transcription of the GFP gene contributes to growth inhibition, however, synthesis of the GFP protein in LB medium is without impact on cell growth.

### Recombinant protein folding associated problems contribute to growth inhibition

During induction in defined glucose-supplemented mineral salt medium, however, cells carrying the GFP encoding expression vector with the intact promoter region as well as the RBS exhibited a higher growth inhibition than those cells carrying the plasmid with intact promoter region but without RBS (Fig. 5b). Again, SDS-PAGE analyses revealed the presence of GFP in induced cells carrying the production vector with intact promoter region and RBS but not in those induced cells transformed with the expression where the RBS was removed (Fig. 6). Also, real-time PCR analysis proved the presence of GFP mRNA in both types of transformed cells (Fig. 7). Thus, synthesis of recombinant GFP protein in defined glucose-supplemented mineral salt medium augments the recombinant gene transcription mediated growth inhibition.

SDS-PAGE analysis of the soluble and insoluble cell fraction of *E. coli* BL21(DE3) producing either hFGF-2 or GFP on complex Luria–Bertani or glucose-supplemented mineral salt medium revealed that on both types of media the majority of hFGF-2 aggregates into inclusion bodies (Fig. 3). GFP, on the other hand, does not form aggregates during growth on Luria–Bertani medium (Fig. 5). However, during production on glucose-supplemented mineral salt medium also GFP aggregates partly into inclusion bodies although the majority of the protein is still found in the soluble cell fraction (Fig. 6). These findings show that transcription of the recombinant gene clearly contributes to the metabolic burden but recombinant protein translation only if it is connected of protein folding associated problems, e.g. formation of inclusion bodies.

### Growth inhibition also caused by induction of the “empty” expression vector

**Fig. 8:**
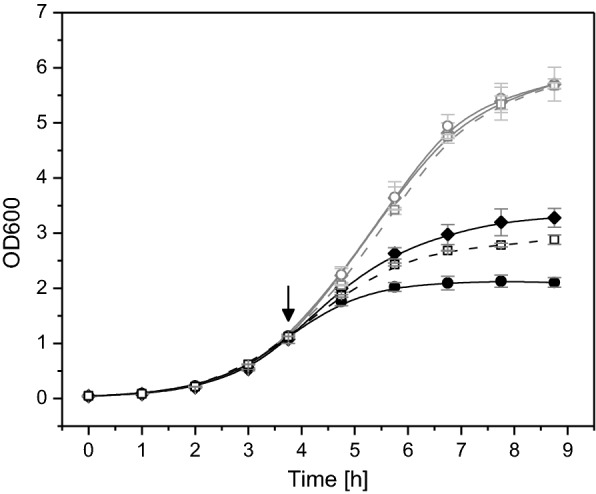
Growth properties of *E. coli* BL21 (DE3) carrying the “empty” pET-28c expression vector. Cells carrying the original “empty” pET-28c expression vector with promoter region and RBS (circles) and the “empty” vector with promoter region but without RBS (diamonds) were grown in shake flask cultures in Luria–Bertani medium. Growth of cells carrying the original expression vector encoding hFGF-2 with promoter region and with RBS is revisited for a better comparison (broken lines, open squares). Black symbols always correspond to IPTG induced cultures and gray symbols to cultures where IPTG addition was omitted. The time-point of IPTG addition to a final concentration of 0.5 mM is indicated by an arrow. Data are presented as mean ± SD, n = 4

Finally, the growth properties of *E. coli* BL21(DE3) carrying the “empty” expression vector pET28c or pET29c were analyzed. Short transcripts with a RBS are encoded downstream of the T7 promoter (pET28c: 225 bp encoding a protein of 8.3 kDa and pET29c: 225 bp encoding a protein of 8.5 kDa). Surprisingly, both transformants showed a strong growth inhibition upon IPTG induction even stronger than the hFGF-2 producing stain (Fig. 8, data only shown for pET28c). SDS-PAGE analysis of induced cells revealed a protein at the expected position in *E. coli* BL21(DE3):pET29c mainly found in the insoluble cell fraction (data not shown). However, no protein band was found in induced *E. coli* BL21(DE3):pET28c at the expected molecular mass of 8.3 kDa (data not shown) suggesting stalled ribosomes or proteolytic degradation of the encoded peptide. Cells carrying pET28c without RBS but intact promoter region still showed strong growth inhibition upon IPTG addition but not as strong as the original pET28c vector with RBS (there is no additional RBS present in the short transcript) (Fig. 8). These data show that also short transcripts originating from “empty” expression vectors cause growth inhibition which can be intensified through corresponding downstream-processes of transcription (e.g. stalled ribosomes, folding problems of the translated protein peptide involving aggregation, degradation and the accompanying heat shock response).

## Discussion

Reduction of the recombinant protein production associated metabolic burden is generally accomplished by decreasing the expression of the recombinant gene. A sometimes detrimental growth inhibition with low levels of the target protein can be overcome, for example, by replacing multicopy plasmids through chromosomal integration of the target gene [8, 9] or by utilizing strains/constructs with reduced target gene promoter activity [13, 17, 18]. All these attempts reduce recombinant gene expression, alleviate the metabolic burden and at the same time increase the target protein amount. These findings suggest that not the amount of the recombinant protein but rather recombinant gene transcription, the mRNA level or even the speed of translation are the major sources for the metabolic burden. Our data show that transcription or the transcript contributes to the metabolic burden but not protein translation itself. Recombinant protein synthesis appears to add only to the metabolic burden if it is connected to protein folding related problems during production, e.g. inclusion body formation, proteolytic degradation and connected cell responses such as heat shock responses. In the best case, e.g. high-level production of GFP on LB medium, translation of the transcript into a properly folded protein does not show any effect on cell growth. This proves that elevated translation of a useless protein—a highly energy consuming process—does not have a compulsatory negative effect on host cell growth. But a negative effect is clearly exerted through elevated transcription or elevated transcript levels. On the other hand, translation or those processes following translation can enhance the transcription related growth inhibition. In this case it might not always be possible to clearly assign this additional inhibitory effect to translation (e.g. stalled ribosomes [19]) or to processes following translation of the target protein e.g. inclusion body formation, proteolytic degradation and/or connected cell responses such as heat shock responses. Moreover, the effect can also be combinatorial, e.g. translation is too fast for a slow folding protein leading to the formation of inclusion bodies, and subsequently to an enhanced growth inhibition. This is presumably the case for hFGF-2. hFGF-2 is a slow folding protein [20] that readily forms inclusion bodies and elicits a strong heat-shock response during rapid production in batch cultures [16] this way most likely increasing the transcription related growth inhibition.

After attributing the recombinant protein production related “metabolic burden” originally more to protein synthesis related problems more evidence is nowadays accumulating that transcription or the transcript itself is the major factor of the recombinant protein production related growth inhibition. Recent studies on the production of proteins with synonymous codons revealed that some codons, encoding the same amino acid, were inhibiting cell growth more severely than others [12, 13]. It was also shown that the toxicity of certain gene variants was only dependent on transcription but independent on protein translation [13]. Moreover, it was found that the toxicity of certain mRNA sequences could not be related to plasmid abundance nor to the abundance of the encoded mRNA [13]. Mittal et al. concluded that toxic RNA might interact with essential cellular components and interfere with their normal function through a mechanism not yet known [13]. Our findings are in line with the observation that certain mRNA elements impose a stronger metabolic burden than others and additionally show that excessive transcription or elevated amounts of recombinant mRNA in general lead to growth retardation although not necessarily to severe growth inhibition.

## Materials and methods

### Strain and plasmids

*Escherichia coli* BL21 (DE3) (Novagen, Germany) harboring the plasmids pET-29c-hFGF-2 [15] and pET-28c-His6-GFP [21] were used for the production of hFGF-2 and GFP, respectively. Both genes are codon-optimized and their sequences given in Knoerzer et al. [22] and Crameri et al. [23]. The codon adaptation index (CAI) was determined as 0.79 for hFGF-2 and 0.65 for GFP [24]. The modified plasmids with deletions of RBS site and/or T7 promoter region (Table 1) were constructed using Q5^®^ Site-Directed Mutagenesis Kit (New England Biolabs, USA). NEBaseChanger™ tool (New England Biolabs, USA) was used to design primer sequences and infer annealing temperatures. The detailed experimental conditions were according to manufacturer’s instructions. Sequences of parental and mutant plasmids were confirmed by Sanger sequencing (Microsynth Seqlab, Germany).

**Table 1:** Plasmids

Plasmids	Features
pET-28c/pET-29c	Novagen, Germany
pET-28cΔRBS	Mutant plasmid with deletion of all RBSs (AAGGAG) between T7 promoter and T7 terminator
pET-29c-hFGF-2	Parental plasmid for the production of hFGF-2 [15]
pET-29c-hFGF-2 ΔRBS	Mutant plasmid with deletion of all RBSs (AAGGAG) between T7 promoter and T7 terminator
pET-29c-hFGF-2 ΔT7	Mutant plasmid with deletion of T7 promoter region (TAATACGACTCACTATAGG)
pET-29c-hFGF-2 ΔRBS & ΔT7	Mutant plasmid with deletion of all RBSs (AAGGAG) between T7 promoter and T7 terminator as well as T7 promoter region (TAATACGACTCACTATAGG)
pET-28c-His6-GFP	Parental plasmid for the production of GFP [21]
pET-28c-His6-GFP ΔRBS	Mutant plasmid with deletion of all RBSs (AAGGAG) between T7 promoter and T7 terminator

### Medium and cultivation conditions

The composition of Luria–Bertani (LB) broth was as follows: 10 g L^−1^ tryptone, 5 g L^−1^ yeast extract, and 5 g L^−1^ NaCl. The pH was adjusted to pH 7 by NaOH before autoclaving. For solidification, 15 g L^−1^ agar were added. The composition of the defined glucose-supplemented mineral salt medium (Defined Non-inducing Broth (DNB) [21]) was as follows: 12 g L^−1^ glucose·H_2_O, 1.2 g L^−1^ MgSO_4_·7H_2_O, 4 g L^−1^ (NH_4_)_2_HPO_4_, 13.3 g L^−1^ KH_2_PO_4_, 1.7 g L^−1^ citric acid·H_2_O, 100.8 mg L^−1^ Fe(III) citrate, 2.1 mg L^−1^ Na_2_MoO_4_·2H_2_O, 1.4 mg L^−1^ CoCl_2_, 12.3 mg L^−1^ MnCl_2_·2H_2_O, 1.2 mg L^−1^ CuCl_2_, 3 mg L^−1^ H_3_BO_3_, 33.8 mg L^−1^ Zn(CH_3_COOH)_2_·2H_2_O, 14.1 mg L^−1^ Titriplex III. The pH was adjusted at pH 6.8 by NaOH. Details of medium preparation are given elsewhere [21]. For plasmid maintenance, 50 mg L^−1^ kanamycin were added to the medium.

Precultures were prepared essentially as described previously [21]: briefly, a single colony from LB agar plate was transferred to LB medium after overnight incubation at 30 °C. The cultures were shaken at 30 °C overnight to inoculate the LB main cultures or at 30 °C for 6 h to inoculate the DNB overnight pre-cultures. DNB pre-cultures were used to inoculate the DNB main cultures. Shake flask cultivations were carried out using 1.8 L Fernbach flasks with three baffles containing 200 mL medium at 30 °C and 180 rpm or 500 mL Erlenmeyer flasks containing 50 mL medium at 30 °C and 250 rpm using a shaker with an amplitude of 5 cm. During bioreactor batch cultivations temperature, pH, aeration, and agitation speed were set at 30 °C, pH 6.9, 1.5 vvm and 300 rpm, respectively. All cultures were IPTG-induced at mid-exponential phase when the optical density reached OD600 1.0–1.5. All cultivations were carried out at least in quadruplicate if not stated otherwise. Mean values and standard deviation are given.

### Basic analytical procedures and calculations

Cell growth was monitored by measurement of the absorbance at 600 nm (OD600). For glucose analysis, the YSI 2300 STAT PlusTM glucose and lactate analyzer (YSI Life Sciences, USA) was used. Off-gas analysis was performed in bioreactor cultures using the BlueInOne gas analyzer (BlueSens, Germany). The carbon dioxide and oxygen transfer rates were calculated as described previously [25]. The dissolved oxygen concentration was determined using a polarographic dissolved oxygen sensor (Mettler Toledo, Germany). For preparation of cell extracts and determination of soluble and insoluble product fractions, cells were disrupted by BugBuster™ Protein Extraction Reagent (Novagen, USA) with rLysozyme and Benzonase according to manufacturer’s instructions. Soluble and insoluble cell fractions were separated by centrifugation at 17,000*g* and 4 °C for 30 min. SDS-PAGE analysis was performed in the Mini-PROTEAN Tetra Cell (Bio-Rad, USA) according to standard procedures and manufacturer’s instructions. After electrophoresis, proteins were visualized by colloidal Coomassie G-250 staining [26].

### RNA extraction

Cultivation samples were transferred to centrifuge tubes which were prefilled with an equal volume of inhibition solution (5% H_2_O saturated phenol in ethanol). Cells were pelleted by centrifugation (17,000*g* and 4 °C for 5 min) and the supernatant was removed. The pellets were resuspended in half the original volume of RNAlater solution (25 mM sodium citrate, pH 5.2, 10 mM EDTA, 700 g L^−1^ ammonium sulfate) and stored at − 70 °C. Before hot phenol RNA extraction, cells stored in RNAlater solution were centrifuged at 17,000*g* and 4 °C for 10 min. After discarding the supernatant, the pellets were resuspended in 900 µL DEPC-treated H_2_O with 400 U mL^−1^ rLysozyme (Merck, Germany). After addition of 90 µL 10% SDS, samples were incubated at 800 rpm and 27 °C for 10 min and subsequently heated at 65 °C for 2 min. Afterwards, 100 µL 1 M sodium acetate, pH 5.2 and 1 mL H_2_O saturated phenol (pH < 7) were added to the samples. Samples were inverted several times to mix and incubated at 65 °C for 7 min. Every 40 s, the samples were inverted several times. After centrifugation at 17,000*g* and 4 °C for 15 min, the aqueous top layer was transferred to a new tube. The same volume of chloroform was added. After complete mixing and subsequent centrifugation at 17,000*g* and 4 °C for 10 min, the aqueous top layer was again transferred to a new tube. To precipitate RNA, 100 µL 5 M NaCl and 1–2 mL cold ethanol (− 20 °C) were added. After centrifugation at 17,000*g* and 4 °C for 20 min, the RNA precipitate was washed once with 75% ethanol, centrifuged again at 17,000*g* and 4 °C for 20 min and the supernatant was discarded. Air-dried RNA pellets were resuspended in nuclease-free water and stored at − 20 °C.

### cDNA synthesis

27 μL reaction mixture containing 1 μg of total RNA and 0.375 μg of random hexamer primer was heated to 65 °C for 5 min to melt secondary structure within the template. After cooling immediately on ice for 1 min, 4 μL dNTPs (10 mM each), 1 μL M-MLV reverse transcriptase (Promega, USA) and 8 μL 5× Reaction Buffer were added to the reaction mixture. The reverse transcription was carried out at 37 °C for 1 h. The synthesized cDNA was stored at − 20 °C.

### Quantitative real-time PCR

qPCR primers (Table 2) were designed using Eurofins’s online PCR primer design tools (https://www.eurofinsgenomics.eu/en/ecom/tools/pcr-primer-design/, Eurofins Genomics, Germany). qPCR was performed in an IQ5 Multicolor Real-Time PCR Detection System (Bio-Rad, USA) using GoTaq^®^ qPCR Master Mix (Promega, USA). Each 25 μL reaction mixture contained 12.5 μL of 2× qPCR Master Mix, 0.5 μL cDNAs and 0.5 μL of each primer. Amplification conditions were 3 min at 95 °C, followed by a three-steps cycle of denaturation at 95 °C for 30 s, annealing at 57.5 °C for 30 s and extension at 72 °C for 30 s for a total of 50 cycles. All qPCR reactions were carried out in triplicate and mean values are given. 16S ribosomal RNA (*rrsH*) was used as the internal standard. Expressions of translation elongation factor EF-Tu (*tufA*), hFGF-2 and GFP genes were normalized to *rrsH*, whose relative gene expression was artificially defined as 10,000 for a better comparison.

**Table 2:** Primers for qPCR

Gene	Primer sequence 5′–3′	Tm (°C)	Amplicon (bp)
*rrsH*	Fw: TCAGCGGGGATGAAGGGAGTAAAG	58.0	209
	Rev: GCCAGTATCAGATGCAGTTCCCAG	57.4	
*tufA*	Fw: TTCTTCAAAGGCTACCGTCCGC	57.1	211
	Rev: GAACTTTAGCAACAACGCCCGC	58.1	
hFGF-2	Fw: AAGACCCGAAACGTCTGTACTGC	57.1	215
	Rev: GGTAACGCATTTGGAAGCCAGC	57.1	
GFP	Fw: TGGAAAACTACCTGTTCCCTGGC	56.6	198
	Rev: CCTTCAAACTTGACTTCAGCACGC	57.3	

## Data Availability

All data generated or analyzed during this study are included in this published article.
